# *TFAP2B*, *AP-1* and *JAZF1* Expression in Tissues of Papillary Thyroid Carcinoma Patients; Clinical, Pathological and Prognostic Values

**DOI:** 10.31557/APJCP.2020.21.8.2415

**Published:** 2020-08

**Authors:** Abdulwahab A Abuderman, Ola A Harb, Loay M Gertallah, Nahlah Makki Almansour

**Affiliations:** 1 *Department of Basic Medical Sciences, College of Medicine, Prince Sattam Bin Abdulaziz University, Al-Kharj, Saudi Arabia.. *; 2 *Department of Pathology, Faculty of Medicine, Zagazig University, Zagazig, Egypt. *; 3 *Department of General Surgery, Faculty of Medicine, Zagazig University, Zagazig, Egypt. *; 4 *Department of Biology, College of Science, University of Hafr Al Batin, Hafr Al Batin, Saudi Arabia. *

**Keywords:** Papillary thyroid cancer, TFAP2B- AP-1, JAZF1, prognosis, immunohistochemistry

## Abstract

**Objective:**

Transcription factor activating protein 2 B (TFAP2 B) is a transcription factor that regulates many steps of embryogenesis, cell growth, apoptosis and recently oncogenesis and cancer progression. AP-1 is a transcription factor that is a downstream molecule of the MAPK signaling pathway. Juxtaposed with zinc finger gene 1 (*JAZF1*) is a recently detected transforming growth factor which has a role in carcinogenesis. Hence the present study aimed to assess those markers expression in tissues from patients with such cancer correlation their expression with clinic-pathological findings of the tumor and prognostic and follow-up findings of patients.

**Methods:**

We have collected tissue samples from papillary thyroid cancer patients and adjacent non-neoplastic tissues from 80 patients. We assessed the expression of *TFAP2B, AP-1* and *JAZF*1 using immunohistochemistry.

**Results:**

Expression of *TFAP2B* was positively associated with lymph nodes metastases (p=0.003), distant metastases (p=0.002), recurrence of the tumor (p=0.002), unfavourable disease-free survival rate (p=0.003). *AP-1* expression is positively associated with advanced stage (p=0.002), presence of extra-thyroid invasion (p=0.005), recurrence of the tumor (p=0.005), unfavorable disease-free survival rate (p=0.01). *JAZF1* expression is negatively associated with huge tumor size (0.023), vascular invasion (p=0.007) and unfavorable overall survival rate (p=.030).

**Conclusion:**

High expression levels of *TFAP2B* and *AP-1* and low expression levels of JAZF1 were associated with unfavourable pathological, prognostic parameters and dismal patient’s outcome.

## Introduction

Thyroid cancer is considered the most prevalent endocrine system cancers that continued rising worldwide in the past decades (Xiao et al., 2019). Papillary carcinoma of the thyroid formed about 74–80% of all thyroid malignancies (Huang et al., 2019). Papillary thyroid cancer has a favourable patients outcome and better patients survival, but it was found that some patients might have more aggressive phenotypes with lack of response to the currently used therapies with high patients morbidity and mortality (Fu et al., 2019). Therefore, identifying new mechanisms of cancer progression and novel molecular biomarkers might help in the identification of patients having a higher liability of papillary carcinoma of the thyroid recurrence and progression. Transcription factor activating protein 2 B (TFAP2 B) is a transcription factor that regulates many steps of embryogenesis, cell growth, apoptosis and recently oncogenesis and cancer progression (Xu et al., 2017, Fu et al., 2019). AP-1 is a transcription factor that is a downstream molecule of the MAPK signaling pathway, which is incriminated in controlling many cellular processes as cell growth, apoptosis and proliferation (Li et al., 2016). Disturbances in the expression of *AP-1* were linked to tumor transformation, progression, invasion, metastases and angiogenesis (Tewari et al., 2018). Juxtaposed with zinc finger gene 1 (*JAZF1*) is a recently detected transforming growth factor which has a role in carcinogenesis (Nakajima et al., 2004). As no previous studies have assessed the roles of *TFAP2B, AP-1* and *JAZF1* expression in patients with papillary carcinoma of the thyroid, we aimed to assess *TFAP2B, AP-1* and *JAZF1* expression in tissues from patients with such cancer correlation their expression with clinic-pathological findings of the tumor and prognostic and follow-up findings of patients.

## Materials and Methods

We had collected tissue samples from papillary thyroid cancer patients and adjacent non-neoplastic tissues from 80 patients who were operated in the University’s Teaching Hospitals and other Private Hospitals.

*Inclusion criteria*

Patients with a confirmed diagnosis of papillary thyroid carcinoma, Patients with completed clinical data, Patients who accepted to be included in the study, patients without a history of pre-operative chemotherapy or radiotherapy treatment

*Exclusion criteria*

Patients with incomplete data, patients who have another cancer, patients received any pre-operative treatment; all included patients provided written informed consent to be included in the study. An approval for performing the study was taken from the local ethical committee of the institutional review board of Zagazig University, Faculty of Medicine, Egypt.”

*Immunohistochemistry technique*

The tissues which were collected from the surgical samples have been embedded in a paraffin block, cut at a thickness of 3-μm for performing immunohistochemistry by incubation with primary polyclonal anti; TFAP2B, AP-1 and JAZF1 antibodies at dilution 1:100 (Abcam, Cambridge, UK). 

*Assessment of TFAP2B, AP-1 and JAZF1 expression in stained tissues*

We have assessed both staining extent and intensity; staining extent was estimated on scores from 1-4 (0, negative; 1 less than 10%; 2 from 10 to 50%; 3 from 51 to 80%; 4 more than 80%). The staining intensity was estimated on scores from 1-3 (0, negative staining; 1 represented weak staining; 2, represented moderate staining; 3represented strong staining. The final score of TFAP2B, AP-1 and JAZF1 was reached by multiplying scores of extent and intensity, 2 was the cut point, and ≥ 5 was positive expression while below 5 is negative.

*Statistical analysis*

For data analysis we have used software statistical analysis program SSSP 24.0 (Chicago, USA) was used for data analysis, using; Chi-square test for analysis of differences in protein expression of *TFAP2B, AP-1* and *JAZF1* in collected tissues. Kaplan Meier plots were used to assess patients’ survival. P < 0.05 was considered a statistically significant value.

## Results

We included 80 patients (22 males, 58 females), with their age, ranging from 16 to 70 years. There were 48 patients have cervical lymph node spread, and 26 patients have an extra-thyroid invasion. We included samples from 80 papillary thyroid cancers; 72 of them were diagnosed as having conventional papillary thyroid carcinoma, and 8 of them were diagnosed as having a follicular variant of papillary thyroid carcinoma and 80 samples from adjacent non-neoplastic thyroid tissue of the same patients (Table 1).

*TFAP2B expression in thyroid tissue*

*Immunohistochemistry results*

**Figure 1:**
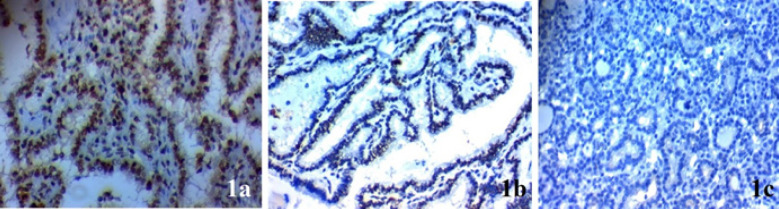
*TFAP2B* Expression in Papillary Thyroid Carcinoma (PTC). (A) High cytoplasmic expression in poorly differentiated PTC of high stage × 400, (B) low cytoplasmic expression in well differentiated PTC of low stage × 400, (C) negative cy

**Figure 2:**
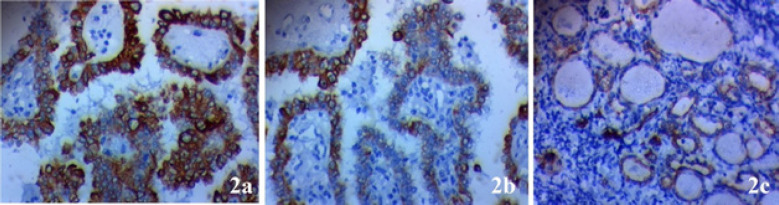
*AP-1 *Expression in Papillary Thyroid Carcinoma (PTC). (A) High nuclear expression in poorly differentiated PTC of high stage × 400, (B) low nuclear expression in well differentiated PTC of low stage × 400, (C) negative nuclear exp

**Figure 3:**
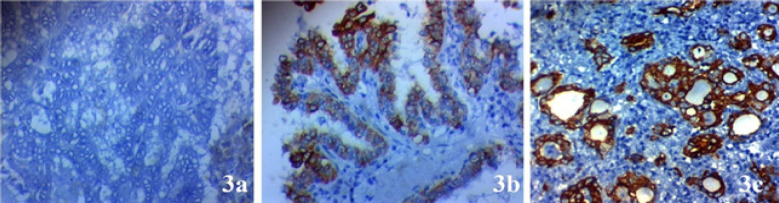
*JAZF1* Expression in Papillary Thyroid Carcinoma (PTC). (A) negative cytoplasmic expression in poorly differentiated PTC of high stage × 400, (B) low cytoplasmic expression in well differentiated PTC of low stage × 400, (C) high cy

Expression of *TFAP2B* was elevated in samples from papillary thyroid carcinoma more than samples from adjacent non-neoplastic thyroid tissue of the same patients (p=0.002).* TFAP2B* expression in papillary thyroid cancer tissues is positively associated with advanced stage, lymph nodes metastases (p=0.003), huge tumor size (p=0.022), presence of extra-thyroid invasion (p=0.005), multifocality of the tumor (p=0.002), vascular invasion (p=0.028), capsular invasion (p=0.004) and distant metastases (p=0.002) (Table 1 and Figure 1-3).

*Patients’ outcome and survival results*

**Figure 4:**
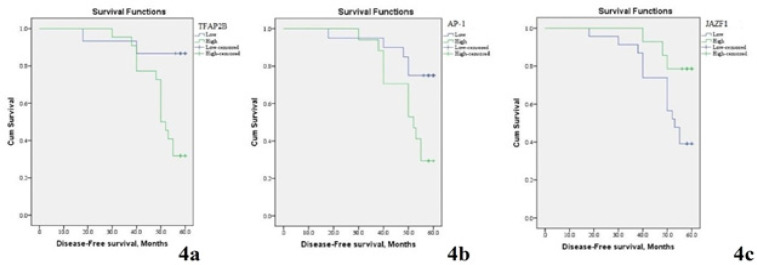
Kaplan Meir Survival Curves of Five-Year Disease Free Survival (DFS) of Included Papillary Thyroid Carcinoma (PTC) Patients. (A) DFS rate stratified according to TFAP2B expression (B) DFS rate stratified according to AP-1 expression, (C) PFS

**Figure 5:**
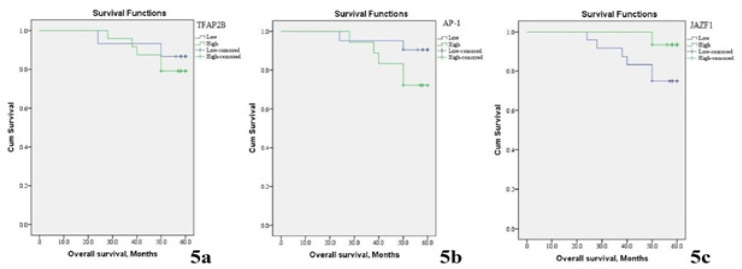
Kaplan Meir Survival Curves of Five-Year Overall Survival Rate (OS) of Included Papillary Thyroid Carcinoma (PTC) Patients. (A) OS rate stratified according to TFAP2B expression (B) OS rate stratified according to AP-1 expression, (C) OS rat

*TFAP2B* expression in papillary thyroid cancer tissues is positively associated with a high incidence of disease progression, recurrence of the tumor (p=0.002), unfavourable disease-free survival rate (p=0.003) (Tables 2-4 and Figures 4-5).

*AP-1 expression in thyroid tissue*

*Immunohistochemistry results*

Expression of AP-1was elevated in samples from papillary thyroid carcinoma more than samples from adjacent non-neoplastic thyroid tissue of the same patients (p=0.005). *AP-1* expression in papillary thyroid cancer tissues is positively associated with advanced stage, lymph nodes metastases (p=0.002), huge tumor size (0.034), presence of extra-thyroid invasion (p=0.005), multifocality of the tumor (p=0.003), vascular invasion (p=0.02), capsular invasion (p=0.022) and distant metastases (p=0.042) (Table 1 and Figure 2)

*Patients’ outcome and survival results*

*AP-1* expression in papillary thyroid cancer tissues is positively associated with a high incidence of disease progression, recurrence of the tumor (p=0.005), unfavourable disease-free survival rate (p=0.01) (Tables 2-4 and Figures 4-5).

*JAZF1 expression in thyroid tissue*

*Immunohistochemistry results*

Expression of *JAZF1* expression was decreased in samples from papillary thyroid carcinoma more than samples from adjacent non-neoplastic thyroid tissue of the same patients (p=0.004).* JAZF1* expression in papillary thyroid cancer tissues is negatively associated with advanced stage (p=0.34), lymph nodes metastases (p=0.005), huge tumor size (0.023), presence of extra-thyroid invasion (p=0.045), multifocality of the tumor (p=0.036), vascular invasion (p=0.007) and capsular invasion (p=0.037) (Table 1 and Figure 3)

*Patients’ outcome and survival results*

*JAZF1* expression in papillary thyroid cancer tissues is negatively associated with a high incidence of disease progression, recurrence of the tumor (p=0.031), unfavourable disease-free survival rate and unfavourable overall survival rate (p=0.030) (Tables 2-4 and Figures 4-5). There was a positive association between *TFAP2B* and *AP-1* expression (r=+0.460, p=0.004), inverse association between* TFAP2B* and *JAZF1* expression (r=-0.263, p=0.003) and inverse association between* AP-1 *and *JAZF1 *expression (r=-0.645, p=0.005).

**Table 1:** Association of Clinicopathological Features with *TFAP2B, AP-1 *and *JAZF1* Expression in Included Papillary Thyroid Carcinoma Patients

		TFAP2B				p	AP-1				p	JAZF1				p
		Low		High			Low		High			Low		High		
		N=30		N=50			N=42		N=38			N=50		N=30		
Age.Group	<40y	12	40.00%	28	56.00%	0.337	14	33.30%	26	68.40%	0.033	30	60.00%	10	33.30%	0.002
	=>40y	18	60.00%	22	44.00%		28	66.70%	12	31.60%		20	40.00%	20	66.70%	
Sex	M	10	33.30%	12	24.00%	0.422	12	28.60%	10	26.30%	0.233	14	28.00%	8	26.70%	0.827
	F	20	66.70%	38	76.00%		30	71.40%	28	73.70%		36	72.00%	22	73.30%	
Histo- pathological subtype	PTC conv	28	93.30%	44	88.00%	0.346	40	95.20%	32	84.20%	0.546	44	88.00%	28	93.30%	0.586
	PTC FV	2	6.70%	6	12.00%		2	4.80%	6	15.80%		6	12.00%	2	6.70%	
Stage	I	12	40.00%	2	4.00%	0.003	14	33.30%	0	0.00%	0.002	6	12.00%	8	26.70%	0.034
	II	14	46.70%	8	16.00%		18	42.90%	4	10.50%		8	16.00%	14	46.70%	
	III	2	6.70%	26	52.00%		4	9.50%	24	63.20%		24	48.00%	4	13.30%	
	IV	2	6.70%	14	28.00%		6	14.30%	10	26.30%		12	24.00%	4	13.30%	
Tumor size	<4cm	26	86.70%	24	48.00%	0.022	34	81.00%	16	42.10%	0.034	24	48.00%	26	86.70%	0.023
	=>4cm	4	13.30%	26	52.00%		8	19.00%	22	57.90%		26	52.00%	4	13.30%	
Surgery (thyroidectomy)	Total	12	40.00%	28	56.00%	0.03	20	47.60%	20	52.60%	0.031	28	56.00%	12	40.00%	0.094
	Subtotal	10	33.30%	4	8.00%		12	28.60%	2	5.30%		4	8.00%	10	33.30%	
	Total +BND	8	26.70%	16	32.00%		8	19.00%	16	42.10%		18	36.00%	6	20.00%	
	lobectomy	0	0.00%	2	4.00%		2	4.80%	0	0.00%		0	0.00%	2	6.70%	
Multifocality		6	13.30%	39	60.00%	0.002	8	19.00%	26	68.40%	0.003	28	56.00%	6	20.00%	0.036
LN involvement		8	26.70%	40	80.00%	0.003	12	28.60%	36	94.70%	0.002	38	76.00%	10	33.30%	0.005
Vascular invasion		4	6.70%	18	36.00%	0.028	6	14.30%	14	36.80%	0.02	16	32.00%	4	13.30%	0.007
Capsular invasion		4	6.70%	28	56.00%	0.004	8	19.00%	22	57.90%	0.022	24	48.00%	6	20.00%	0.037
Extrathyroid extension		4	6.70%	24	48.00%	0.005	6	14.30%	20	52.60%	0.032	22	44.00%	4	13.30%	0.045
Distant metastasis		4	6.70%	14	28.00%	0.002	6	14.30%	10	26.30%	0.042	12	24.00%	4	13.30%	0.414

**Table 2:** The Outcome of Patients in Relation to *TFAP2B, AP-1* and *JAZF1* Expression in Included Papillary Thyroid Carcinoma Patients

	TFAP2B				p	AP-1				p	JAZF1				p
	Low		High			Low		High			Low		High		
	N=30		N=50			N=42		N=38			N=50		N=30		
Response															
PD	0	0.00%	0	0.00%	0.03	0	0.00%	0	0.00%	0.004	0	0.00%	0	0.00%	0.0872
SD	0	0.00%	2	4.00%		0	0.00%	2	5.30%		2	4.00%	0	0.00%	
PR	0	0.00%	4	8.00%		2	4.80%	2	5.30%		2	4.00%	2	6.70%	
CR	30	100.00%	44	88.00%		40	95.20%	34	89.50%		46	92.00%	28	93.30%	
Overall Response															
NoCR	0	0.00%	6	12.00%	0.049	2	4.80%	4	10.50%	0.479	4	8.00%	2	6.70%	0.347
CR	30	100.00%	44	88.00%		40	95.20%	34	89.50%		46	92.00%	28	93.30%	
Recurrence															
No	26	86.70%	14	28.00%	0.002	30	71.40%	10	26.30%	0.005	18	36.00%	22	73.30%	0.031
Yes	4	13.30%	30	60.00%		10	23.80%	24	63.20%		28	56.00%	6	20.00%	
Outcome															
Censored	26	86.70%	40	80.00%	0.041	38	90.50%	28	73.70%	0.053	38	76.00%	28	93.30%	0.162
Died	4	13.30%	10	20.00%		4	9.50%	10	26.30%		12	24.00%	2	6.70%	

**Table 3:** Survival Analysis in Whole PTC Group and in Relation to *TFAP2B, AP-1* and *JAZF1* Expression in Included Papillary Thyroid Carcinoma Patients

			Survival Time (Months)				Survival rate	P
			Means ±SE (95% CI)		Median±SE (95% CI)		(%)	
5-year Overall Survival	TFAP2B	Low	56.9 ± 2.4	(52.3-61.56)	NR		86.7	0.571
		High	56.1 ± 1.7	(52.68-59.49)	NR		79.2	
	AP- 1	Low	57.8 ± 1.7	(54.45-61.17)	NR		90.5	0.149
		High	54.8 ± 2.2	(50.4-59.16)	NR		72.2	
	JAZF1	Low	54.6 ± 2.2	(50.34-58.83)	NR		75	0.142
		High	59.3 ± 0.6	(58.07-60.6)	NR		93.3	
	Overall		56.4 ± 1.4	(53.66-59.16)	NR		82.1	
5-year Disease-Free Survival	TFAP2B	Low	55.9 ± 2.9	(50.16-61.57)	NR		86.7	0.003
		High	51 ± 1.8	(47.4-54.51)	50 ± 1.7	(46.72-53.28)	31.8	
	AP-1	Low	55.3 ± 2.3	(50.85-59.75)	NR		75	0.01
		High	50.2 ± 2.2	(45.85-54.5)	52 ± 5.4	(41.51-62.49)	29.4	
	JAZF1	Low	50.5 ± 2.3	(45.95-55.01)	53 ± 3	(47.13-58.87)	39.1	0.03
		High	57 ± 1.6	(53.81-60.19)	NR		78.6	
	Overall		52.9 ± 1.6	(49.72-56.17)	NR		54.1	

NR, not reached

**Table 4:** Univariate Cox Regression Analyses of Different Prognostic Factors for Overall Survival

	HR	95.0% CI	P
Age <40 vs >40Y	2.37	0.46-12.19	0.303
Sex M vs F	0.55	0.12-2.44	0.427
Histopathological subtype	1.35	0.16-11.21	0.782
*TFAP2B*, low vs. high	1.59	0.31-8.2	0.58
*AP-1*, low vs. high	3.10	0.6-16.01	0.176
*JAZF1*, low vs. high	0.24	0.03-1.97	0.183
Multifocality	2.08	0.46-9.29	0.339
LN involvement	0.95	0.21-4.25	0.947
Vascular invasion	1.36	0.26-7.01	0.714
Capsular invasion	0.70	0.14-3.59	0.665
Extra-thyroid extension	0.79	0.15-4.05	0.773
Stage	1.45	0.66-3.17	0.357
Distant metastasis	1.92	0.37-9.9	0.435
Surgery	1.45	0.68-3.09	0.332
Tumor size	0.02	0.001-12.5	0.239

HR, Hazard ratio; CI, confidence interval

## Discussion

It is essential to detect novel valid therapeutic target for papillary thyroid cancer mainly for cases resistant to currently used therapies. In our study, we found that *TFAP2B* expression was elevated in cells of papillary thyroid cancer in comparison to adjacent non-neoplastic thyroid tissues which are similar to results of Fu et al., (2019), in papillary thyroid cancer and Fu et al., (2014) in lung cancer. Fu et al., (2019) showed that *TFAP2B* expression in lung cancer tumor tissue samples is markedly elevated in comparison to healthy tissues.

Moreover, Fu et al., (2019), stated that knocking down TFAP2B in cell lines of thyroid cancer reduced viability, proliferation, migration, and invasion of cancer cells. In our study, we clarified that *TFAP2B* higher expression increased the growth of papillary thyroid cancer. That TFAP2B explained these results binds to the promoter of COX-2 in malignant thyroid cells that accelerated cell proliferation and spread so that overexpression of TFAP2B might be rescued by celecoxib which is a COX-2 inhibitor and COX-2 knockdown (Fu et al., 2019).

Additionally, overexpression of TFAP2B stimulated VEGF/PEDF signaling pathway, which has a role in cancer progression. *TFAP2B* high expression stimulated cancer progression through activation of many signaling pathways as; caspase and ERK/p38 in lung cancer (Fu et al., 2014). 

COX-2 inhibitors might be applied in the clinical therapeutic trials against thyroid cancer to decrease its spread and improve patients’ prognosis. Fu et al., (2014), assessed TFAP2B roles in cancer lung progression and stated that it is incriminated in vascularization, progression, metastasis, and cancer recurrence, which is similar to results of previous studies (Karjalainen et al., 2000, Schulte et al., 2008, Tellez et al., 2003). 

Due to relatively few studies regarding the roles of TFAP2B in cancer progression, we assessed the expression of *AP-1*. We showed that there is up-regulation of the expression of *AP-1* in thyroid cancer cells and its expression is related to huge tumor size, Similarly Xiao et al., (2019) who showed that *AP-1* expression in the group of patient with large tumor size is more than patients with smaller tumors. And it is proved that the size of the tumor of papillary thyroid cancer is a good predictor of recurrence of the tumor and dismal outcome (Ito et al., 2012). AP-1 activation stimulated the expression of *VEGF *to the increased proliferation of tumor cells (Daft et al., 2015). Additionally, AP-1 increased tumor invasion and metastasis of through regulating matrix metalloproteinase 9 (MMP-9) through interaction with MMP-9 promoter (Motomura et al., 2018). AP-1 inhibitors might lead to blocking angiogenesis and invasion of cancer cells (Dong et al., 2012).

Moreover, MMP-9 up-regulation degraded the extracellular matrix, which caused malignant invasion and progression (Luo et al., 2017). AP-1 increased MMP9 activity that increased esophageal carcinoma cells invasion and metastasis (Shin et al., 2016). We showed that AP-1 was positively associated with the presence of metastasis to the lymph nodes in papillary thyroid carcinoma patients, which is slightly different from Xiao et al., (2019) who showed no statistically significant differences between the expression of *AP-1* and presence of lymph node spread but they had not clarified the reason.

Different from our findings, Chen et al., (2016) showed a negative association between *AP-1* expression and tumor size. These different results may be attributed to a different number of patient’s different primary antibody used of different methods of assessment. We found a positive association between *TFAP2B* and *AP-1 *expression in papillary thyroid carcinoma patients, but due to a few studies which assessed their expression, we assessed the expression of another biomarker which is *JAZF1*. 

JAZF1 was found to control gluconeogenesis, so, previous studies focused mainly on its role in diabetes and metabolism of lipid (Wei et al., 2018, Jang et al., 2014). Recently, JAZF1 was found to control the biological behaviours of many tumors as endometrial sarcomas and cancer prostate, and it was considered a tumor suppressor (Hodge et al., 2016, Luo et al., 2017, Hazelett et al., 2014). Tumor suppressors have participated in many biological cellular activities as cell proliferation, apoptosis and control of cell cycle (Pospiechet al., 2018, Qiu et al., 2018, Nikbakht et al., 2018, Bai et al., 2018). 

We showed that JAZF1 was down-regulated in papillary thyroid cancer tissues more than adjacent non-neoplastic tissues, which was similar to results of Huang et al., (2019), whose report was the first in exploring tumor suppressor role of JAZF1 in papillary thyroid carcinoma using immunohistochemistry and they stated that expression of* JAZF1* in papillary thyroid carcinoma was decreased in comparison with adjacent thyroid tissues or with nodular goiter.

Huang et al., (2019), showed that *JAZF1* expression is inversely associated with Ki67 labelling index in cancer cells denoting that JAZF1 made its tumor suppressor function by inhibition of cell proliferation and cell cycle arrest. Yuasa et al., (2015) found different results, showed that increased *JAZF1* expression increased cell proliferation and leads to the progression of the cell cycle, which stimulated oncogenesis in muscle. That JAZF1 role explained this variability differs according to the type of tissue. But the exact role of JAZF1 in cancer is still uncertain needing further studies. Up to our knowledge our study is the first to assess the expression of *TFAP2B, AP-1* and J*AZF1* in malignant and non-neoplastic thyroid tissue using immunohistochemistry and evaluation of novel three markers is the strengths of the study. Further studies are needed to focus on the combined effects of these markers using other methods of assessment as gene expression, which performed on a large number of patients.

In conclusion, we demonstrated that expression levels of TFAP2B and AP-1 protein were increased while the expression levels of* JAZF1* were decreased in papillary thyroid carcinoma in comparison with non-neoplastic thyroid tissues using immunohistochemistry. High expression levels of *TFAP2B* and *AP-1* and low expression levels of *JAZF1* were associated with unfavourable pathological, prognostic parameters and dismal patient’s outcome. So the evaluation of tissue protein expression of those three markers might be helpful in the assessment of prognosis of papillary thyroid carcinoma patients.

## Availability of material

The data of the present study are available with the corresponding author.
